# Oxidative Dehydrogenation of Ethane: Superior Nb_2_O_5_-NiO/Ni-Foam Catalyst Tailored by Tuning Morphology of NiO-Precursors Grown on a Ni-Foam

**DOI:** 10.1016/j.isci.2019.09.021

**Published:** 2019-09-17

**Authors:** Zhiqiang Zhang, Guofeng Zhao, Weidong Sun, Ye Liu, Yong Lu

**Affiliations:** 1 Shanghai Key Laboratory of Green Chemistry and Chemical Processes, School of Chemistry and Molecular Engineering, East China Normal University, Shanghai 200062, China; 2 School of Chemistry and Molecular Engineering, East China Normal University, Shanghai 200062, China

**Keywords:** Catalysis, Organic Reaction, Nanomaterials, Nanostructure

## Abstract

Large-scale shale gas exploitation greatly enriches ethane resources, making the oxidative dehydrogenation of ethane to ethylene quite fascinating, but the qualified catalyst with unique combination of enhanced activity/selectivity, enhanced heat transfer, and low pressure drop presents a grand challenge. Herein, a high-performance Nb_2_O_5_-NiO/Ni-foam catalyst engineered from nano- to macroscale for this reaction is tailored by finely tuning the performance-relevant Nb_2_O_5_-NiO interaction that is strongly dependent on NiO-precursor morphology. Three NiO-precursors of different morphologies (clump, rod, and nanosheet) were directly grown onto Ni-foam followed by Nb_2_O_5_ modification to obtain the catalyst products. Notably, the one from the NiO-precursor of nanosheet achieves the highest ethylene yield, in nature, because of markedly diminished unselective oxygen species due to enhanced interaction between Nb_2_O_5_ and NiO nanosheet. An advanced catalyst is developed by further thinning the NiO-precursor nanosheet, which achieves 60% conversion with 80% selectivity and is stable for at least 240 h.

## Highlights

• A series of Nb_2_O_5_-NiO/Ni-foam catalysts are developed for the ODE reaction
• Catalysts are obtained by Nb_2_O_5_ modification of NiO-precursors grown onto Ni-foam
• Thinning NiO-precursors dramatically improves the ethylene selectivity
• Non-selective O_2_^-^ species are markedly reduced with enhanced Nb_2_O_5_-NiO interaction

## Graphical Abstract

**Figure:**
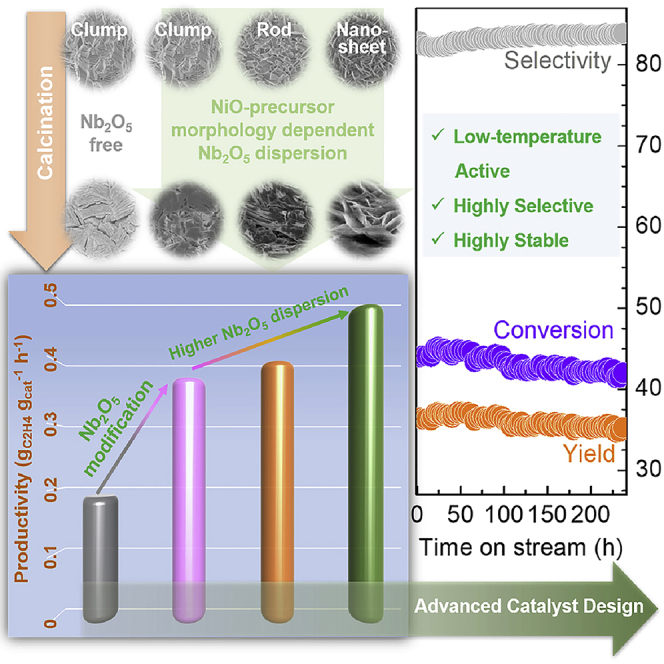

## Introduction

Ethylene (C_2_H_4_) is regarded as the most important petrochemical platform molecule to produce diverse commodity chemicals such as polyethylene, ethylene oxide, vinyl chloride, and polystyrene, with global demand of 153 million tons in 2016 and net added demand of about 5.2 million tons every year (Xu, 2017). Nowadays, its universal production in industry is based on the steam cracking of oil-based naphtha. However, the oil resource is increasingly dwindling, and thus it has turned out to be a hotspot in modern industries to pave the way for efficient and ecofriendly utilization of the nonoil resources (e.g., natural gas, coal, and renewable biomass) to produce ethylene with the aid of effective catalytic processes. Ethane (C_2_H_6_) is abundant in natural gas, and in particular, the shale gas revolution in recent years greatly enriches ethane resources (Sattler et al., 2014). Therefore, ethane-to-ethylene conversion (in terms of oxidative dehydrogenation of ethane [ODE], catalytic dehydrogenation, and steam cracking) tantalizes global enthusiasm. The latter two suffer from their thermodynamic constraints and high operation temperature (>700°C), and the ODE is thus more competitive, benefitting from its oxidative feature that can cast off the thermodynamic limitation and allow lower operation temperature (350°C–550°C) (Heynderickx et al., 2005).

However, controlling the ethylene selectivity for ODE reaction represents the grandest challenge because the excessive oxidation of ethylene to carbon dioxide is thermodynamically and kinetically favorable. Therefore, developing a qualified catalyst with high activity and selectivity is the goal of most efforts for this reaction. To date, various catalysts have been explored (such as alkaline-/rare-earth metal oxides, Mulla et al., 2001, Gaab et al., 2003; noble metals, Fu et al., 2013; and transition metal oxides, Liu et al., 2003, Nakamura et al., 2006), and NiO-based catalysts are the most attractive owing to its low operation temperature, simple preparation, and low cost (Heracleous and Lemonidou, 2006, Heracleous and Lemonidou, 2010, Savova et al., 2010, Zhu et al., 2012). However, NiO alone mainly yields carbon dioxide due to the large amount of electrophilic (unselective) oxygen species (Heracleous and Lemonidou, 2006, Heracleous and Lemonidou, 2010, Savova et al., 2010, Zhu et al., 2012). Many kinds of oxides were doped into NiO to tune the oxidative properties of oxygen species. Lemonidou et al. explored a series of alter-valent cations such as Li, Mg, Al, Ga, Ti, Ta, and Nb (Heracleous and Lemonidou, 2010), and the unselective oxygen amount on NiO surface declines along with the increase in dopant cations' valence. Accordingly, the Nb_2_O_5_-doped catalyst offers the highest ODE performance such as 78% ethylene selectivity and 33% ethane conversion at 350°C (Savova et al., 2010). They further proposed that Nb doping into NiO lattice by filling the cationic vacancies on defective non-stoichiometric NiO surface and/or substituting Ni atoms reduces the amount of unselective oxygen (Zhu et al., 2012, Heracleous and Lemonidou, 2006). However, such Nb_2_O_5_-NiO catalysts suffer from poor stability due to their sintering deactivation (Heracleous and Lemonidou, 2006, Heracleous and Lemonidou, 2010, Savova et al., 2010, Zhu et al., 2012).

Despite the above-mentioned interesting advances, the real-world use of these catalysts still remains a challenge as their poor thermal conductivity is detrimental to rapid dissipation of reaction heat released in this strongly exothermic ODE reaction (ΔH = −104 kJ mol^−1^), which causes severe hotspots in the catalyst bed and therefore leads to the ethylene excessive oxidation while releasing more heat. Recently, the development of structured catalyst based on the monolithic metal-foam has been attracting great interest in heterogeneous catalysis because of the intensified heat transfer, which is favorable to tailor catalysts for strongly exothermic reactions (Chen et al., 2019, Zhao et al., 2016, Zhang et al., 2018a, Zhang et al., 2018b). However, the main issue is how to make these promising metal-foam qualified catalysts, or more concretely, how to fabricate the highly active and selective NiO-based nanocomposites onto foam surface.

Herein, we demonstrate the remarkable improvement of the Nb_2_O_5_-NiO/Ni-foam catalyst performance for ODE reaction, by finely tuning the Nb_2_O_5_-NiO interaction by morphology-controllable growth of NiO-precursors onto Ni-foam.

## Results

### Synthesis, Morphology, and Structural Features of the Ni-Foam Structured NiO-Precursor and Catalysts

**Figure 1:**
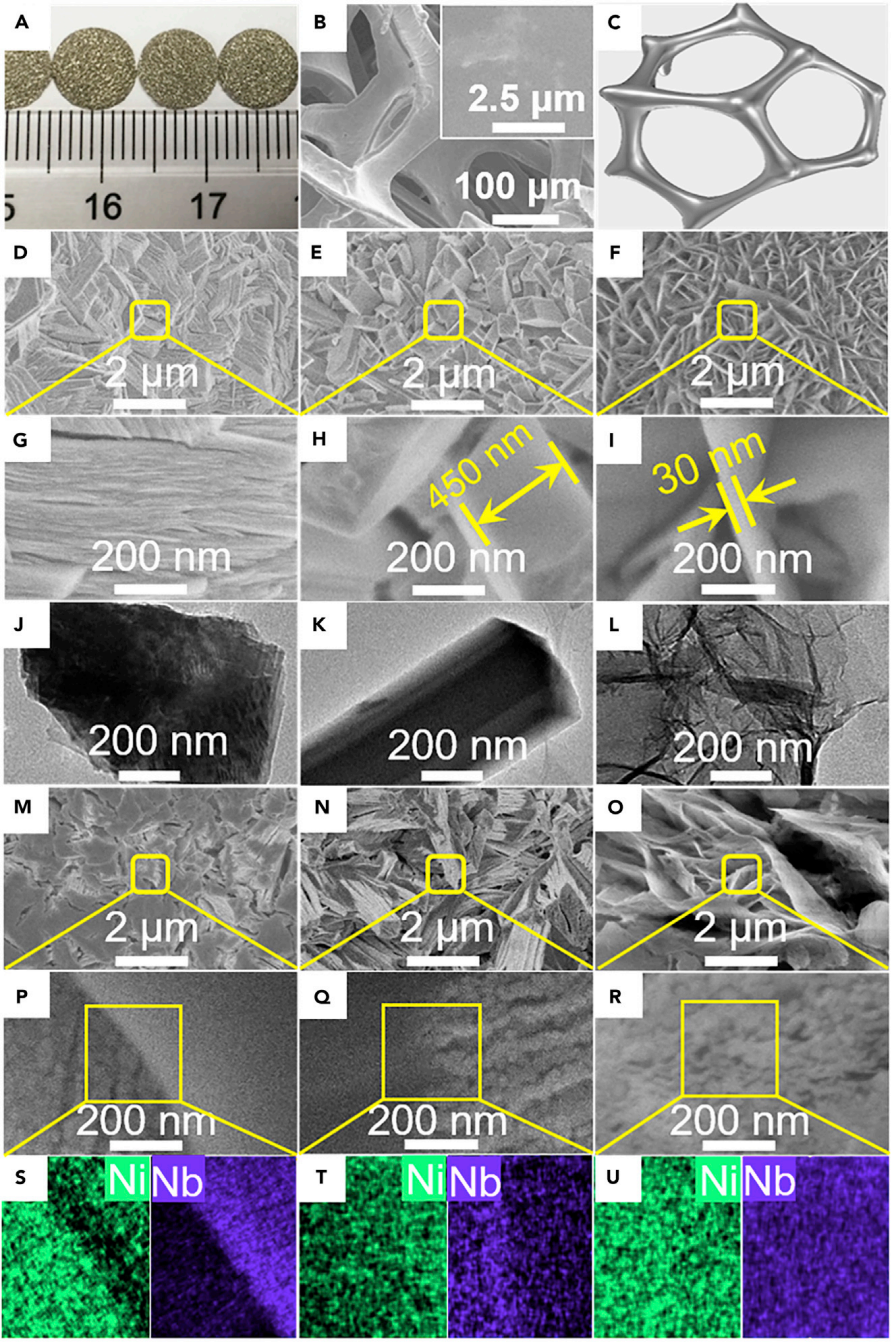
The Structure and Morphological Features of Various NiO-Precursors and Nb_2_O_5_-NiO/Ni-Foam Catalysts (A–C) (A) Optical photograph, (B) scanning electron microscopic (SEM) image, and (C) schematic illustration of network of the pristine Ni-foam. (D, G, and J) (D and G) SEM and (J) transmission electron microscopic (TEM) images of Ni(OH)_2_/Ni-foam. (E, H, and K) (E and H) SEM and (K) TEM images of NiC_2_O_4_/Ni-foam. (F, I, and L) (F and I) SEM and (L) TEM images of Ni-Tp/Ni-foam. (M, P, and S) (M and P) SEM and (S) energy-dispersive X-ray (EDX) images of Nb_2_O_5_-NiO/Ni-foam-C. (N, Q, and T) (N and Q) SEM and (T) EDX images of Nb_2_O_5_-NiO/Ni-foam-R. (O, R, and U) (O and R) SEM and (U) EDX images of Nb_2_O_5_-NiO/Ni-foam-NS.

First, three kinds of NiO-precursors with different morphologies (i.e., clump for Ni(OH)_2_, rod for NiC_2_O_4_, nanosheet for nickel terephthalate (Ni-Tp), identified by X-ray diffracxtion [XRD] in Figure S1) were controllably and endogenously grown onto a Ni-foam (100 pores per inch). Against the smooth surface of Ni-foam (Figures 1A–1C), clearly, the *in situ* growth of three morphology-different NiO-precursor layers on the foam struts succeeds clump with dense stacking for Ni(OH)_2_ layer by ammonia evaporation method (Figures 1D, 1G, and 1J), rod with diameter of about 450 nm for NiC_2_O_4_ layer by hydrothermal method (Figures 1E, 1H, and 1K), and nanosheet of thickness 30 nm for Ni-Tp layer by solvothermal method (Figures 1F, 1I, and 1L). Moreover, unlike the dense layer feature of the Ni(OH)_2_ clump and NiC_2_O_4_ rod, the Ni-Tp nanosheets stand upright and irregularly cross-link each other to form honeycomb-like porous layer. Not surprisingly, the Ni-Tp/Ni-foam delivers a specific surface area (SSA) of 6.3 m^2^ g^−1^ much higher than 1–2 m^2^ g^−1^ for the Ni(OH)_2_/Ni-foam and NiC_2_O_4_/Ni-foam (Table 1).

**Table 1:** Physicochemical Characteristics of the Ni-Foam Structured Catalysts

Catalyst	NiO Loading (wt. %)^a^	NiO Average Size (nm)^b^	Specific Surface Area (m^2^ g^−1^)^c^	NiO Lattice Constant (Å)	TOF^d^
Ni(OH)_2_/Ni-foam	–	–	1.5	–	–
NiC_2_O_4_/Ni-foam	–	–	2.1	–	–
Ni-Tp/Ni-foam	–	–	6.3	–	–
NiO/Ni-foam-C	21.4	19.6	8.7	4.1767	0.61
NiO/Ni-foam-R	21.5	20.3	9.3	4.1768	0.64
NiO/Ni-foam-NS	21.1	19.5	10.7	4.1766	0.62
NiO/Ni-foam-F	20.6	20.9	10.1	4.1769	–
Nb_2_O_5_-NiO/Ni-foam-C	21.2	19.3	12.1	4.1753	0.91
Nb_2_O_5_-NiO/Ni-foam-R	20.9	20.4	13.3	4.1751	0.96
Nb_2_O_5_-NiO/Ni-foam-NS	20.8	13.5	20.8	4.1724	0.93
Nb_2_O_5_-NiO/Ni-foam-F	20.5	12.8	30.3	4.1721	0.94
Nb_2_O_5_-NiO/Ni-foam-F^e^	–	14.5	28.7	–	–

a Estimated by H_2_-TPR (Li et al., 2015) according to the reaction: H_2_ + NiO = Ni + H_2_O, given that Nb_2_O_5_ is normally considered to be an irreducible oxide (Zhang et al., 2018a, Zhang et al., 2018b).

b Calculated by Scherrer equation based on NiO (110) plane.

c Measured by N_2_-BET method.

d TOF (turnover frequency) is defined as the amount of ethylene formed per NiO site per hour (the detailed TOF calculations are provided in Supplemental Information and the related results are listed in Table S1); C_2_H_6_ conversion was controlled to be <5% at 300°C.

e The Nb_2_O_5_-NiO/Ni-foam-F after 240 h testing.

Subsequently, niobium ammonium oxalate was wet-impregnated onto the above-obtained Ni(OH)_2_/Ni-foam, NiC_2_O_4_/Ni-foam, and Ni-Tp/Ni-foam at a Nb_2_O_5_ content of 5 wt. % (including the Ni-foam mass), followed by drying overnight and calcining in air at 450°C, to form Ni-foam-structured Nb_2_O_5_-NiO catalysts (Figures 1M–1U). These catalysts are denoted as Nb_2_O_5_-NiO/Ni-foam-C (clump), Nb_2_O_5_-NiO/Ni-foam-R (rod), and Nb_2_O_5_-NiO/Ni-foam-NS (nanosheet), which all possess equivalent NiO content (∼21 wt. %, including Ni-foam mass; Table 1). The NiO and Ni (from Ni-foam) phases are clearly detected by XRD for all three catalysts, whereas no Nb_2_O_5_ diffraction peaks are observed, indicating its high dispersion or amorphous structure (Figure S2) (Liu et al., 2016). Notably, the Ni(OH)_2_-, NiC_2_O_4_-, and Ni-Tp-derived nano-NiO aggregations show well-preserved clump-, rod- and nanosheet-morphologies regardless of Nb_2_O_5_ introduction (Figures 1M–1O). In addition, the Nb_2_O_5_-NiO ensembles show porous feature in association with the thermolysis of their precursors (Figures 1P–1R) thereby leading to a visible increase in their SSA (Table 1).

Interestingly, the Nb_2_O_5_-NiO/Ni-foam-NS achieves an SSA of 20.8 m^2^ g^−1^, much higher than 12–13 m^2^ g^−1^ seen with the other two catalysts (Table 1). The enhanced surface area can be related to the fact that the nanosheet-like morphology of Ni-Tp/Ni-foam not only favors the formation of catalyst with high SSA (see NiO/Ni-foam-NS, Table 1) but also is helpful for highly dispersing Nb_2_O_5_-precursor onto the Ni-Tp nanosheet to hinder the crystallization of NiO during the calcination process (Solsona et al., 2011, Solsona et al., 2012) (Table 1). Not surprisingly, the Nb_2_O_5_-NiO/Ni-foam-NS catalyst provides an average NiO size of 13.5 nm, smaller than that of ∼20 nm for the Nb_2_O_5_-NiO/Ni-foam-C and Nb_2_O_5_-NiO/Ni-foam-R (Table 1). Nevertheless, the NiO/Ni-foam-NS obtained by calcining the Ni-Tp/Ni-foam in air at 450°C offers an average NiO size of ∼20 nm, being compatible to that seen with the ones derived from Ni(OH)_2_/Ni-foam and NiC_2_O_4_/Ni-foam. This observation reveals that Nb_2_O_5_ introduction favors the decomposition of Ni-Tp nanosheets, rather than Ni(OH)_2_-clump and NiC_2_O_4_-rod, to form smaller NiO nanoparticles. Moreover, the Nb_2_O_5_-NiO/Ni-foam-NS achieves more homogeneous NiO-Nb_2_O_5_ composites than the other two catalysts (Figures 1S–1U).

### ODE Reaction Performance

**Figure 2:**
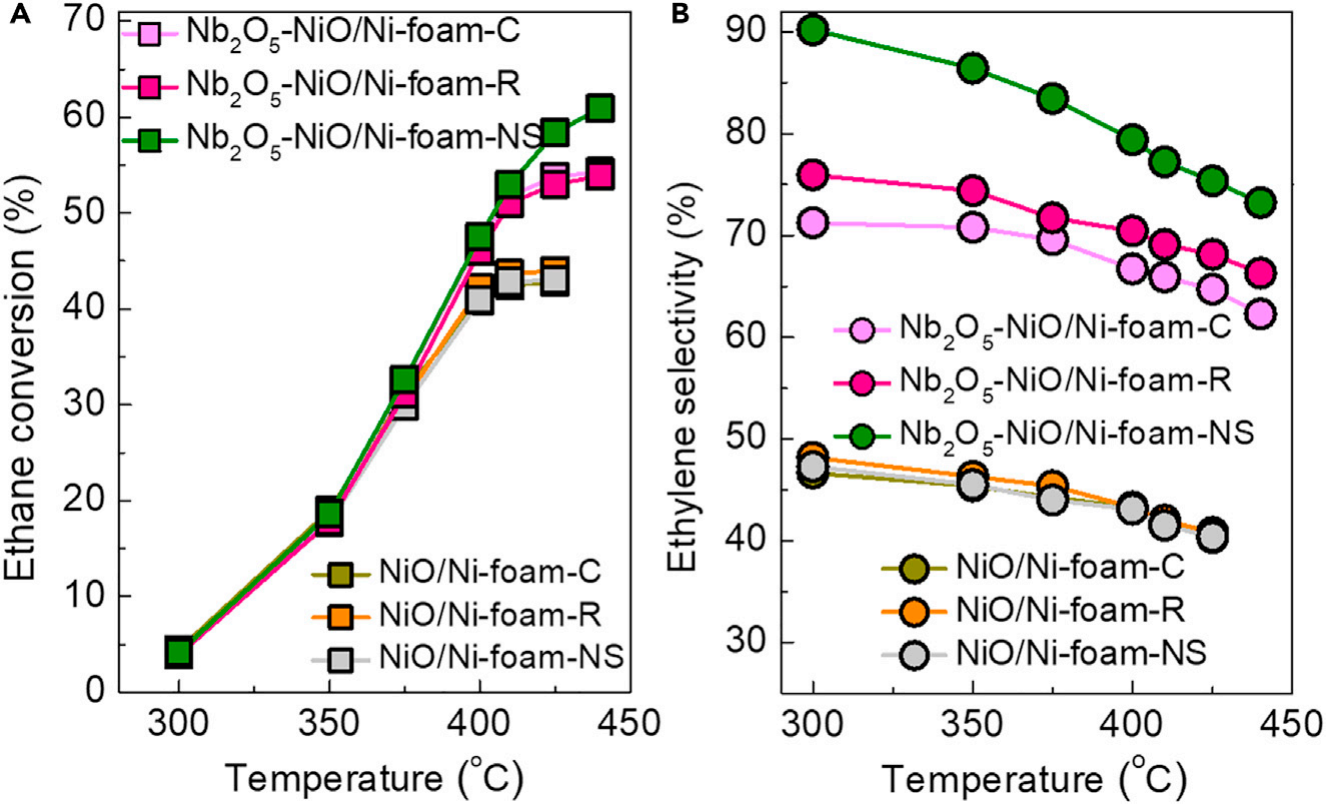
The ODE Performance of the Ni-Foam Structured Catalysts (A and B) Temperature-dependent (A) ethane conversion and (B) ethylene selectivity. Reaction conditions: C_2_H_6_/O_2_/N_2_ of 1/1/8, GHSV of 9,000 cm^3^ g^−1^ h^−1^.

The Nb_2_O_5_ modification dramatically improves the ethylene selectivity and slightly the ethane conversion while leading to a remarkable increase in the turnover frequency (TOF) for ethylene formation from ∼0.62 h^−1^ for the Nb_2_O_5_-free samples to 0.91–0.96 h^−1^ at 300°C (Table 1 and Table S1; the detailed calculation method in the Supplemental Information). As shown in Figure 2, three Nb_2_O_5_-free samples all achieve almost identical ethane conversion and ethylene selectivity in the whole temperature range studied. In contrast, the Nb_2_O_5_-NiO/Ni-foam catalysts exhibit different ODE performance under identical reaction conditions, showing the NiO-precursor morphology dependence; the Nb_2_O_5_-NiO/Ni-foam-NS is obviously superior to the Nb_2_O_5_-NiO/Ni-foam-C and Nb_2_O_5_-NiO/Ni-foam-R catalysts (Figure 2), achieving a 58.4% ethane conversion and 75.4% ethylene selectivity at 425°C. In addition, compared with the very low productivity of only 0.18 g_ethylene_ g_cat._^−1^ h^−1^ over the Nb_2_O_5_-free NiO/Ni-foam catalysts, Nb_2_O_5_ modification gets the ethylene productivity doubled even more. The Nb_2_O_5_-NiO/Ni-foam-NS achieves the highest ethylene productivity of 0.46 g_ethylene_ g_cat._^−1^ h^−1^ (Figure S3).

### Insight into the NiO-Precursor Morphology-Dependent Catalytic Performance

**Figure 3:**
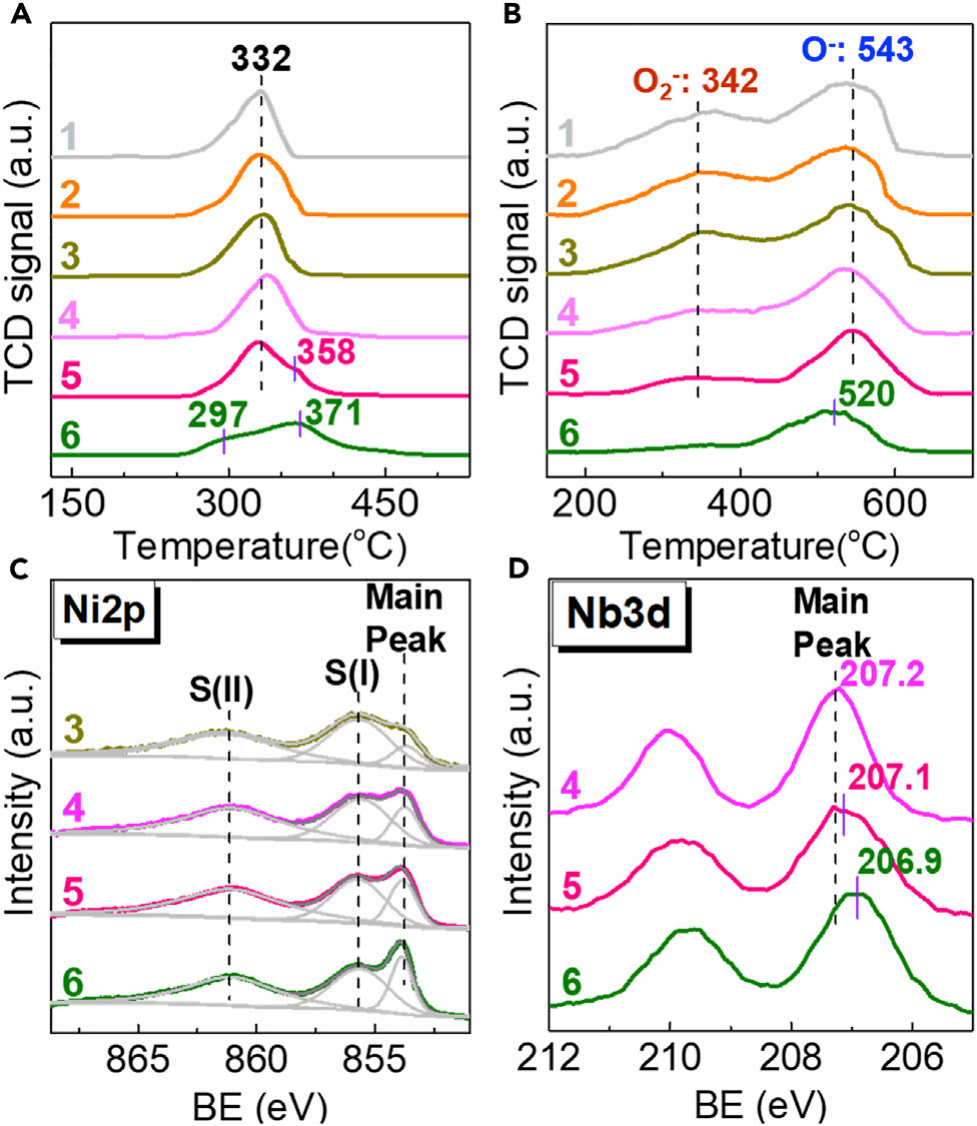
The Characterization Results of the Nb_2_O_5_-NiO/Ni-Foam Catalysts (A–D) (A) H_2_-TPR profiles, (B) O_2_-TPD profiles, and XPS spectra of (C) Ni2p and (D) Nb3d of the catalysts of (1) NiO/Ni-foam-C, (2) NiO/Ni-foam-R, (3) NiO/Ni-foam-NS, (4) Nb_2_O_5_-NiO/Ni-foam-C, (5) Nb_2_O_5_-NiO/Ni-foam-R, and (6) Nb_2_O_5_-NiO/Ni-foam-NS.

To reveal the underlying origin of the NiO-precursor morphology-dependent ODE catalysis on the above Nb_2_O_5_-NiO/Ni-foam catalysts, the amount and type of oxygen species were collaboratively probed by H_2_-temperature-programmed reduction (H_2_-TPR) and O_2_-temperature-programmed desorption (O_2_-TPD) (Zhu et al., 2012, Zhang et al., 2018a, Zhang et al., 2018b). Clearly, whereas the Nb_2_O_5_-free NiO/Ni-foam samples show quite different NiO morphologies (Figure S4), they all possess identical reducibility (by H_2_-TPR) and properties of surface oxygen species (by O_2_-TPD), solidly evidenced by their almost same H_2_-TPR and O_2_-TPD profiles (shape, peak area, and peak temperature; Figures 3A and 3B, profiles 1–3). It is thus not surprising that they achieve NiO-precursor morphology-independent ODE performance (Figure 2). In combining this information with the observation of NiO-precursor morphology dependences of distinct ODE performance after Nb_2_O_5_ modification, it is safe to say that the NiO-Nb_2_O_5_ interaction is sensitive to NiO-precursor morphology, which in nature is responsible for the distinct ODE performance for the Nb_2_O_5_-NiO/Ni-foam catalysts.

Indeed, the reducibility and properties of the surface oxygen species of the Nb_2_O_5_-NiO/Ni-foam catalysts show strong NiO-precursor morphology dependence (Figures 3A and 3B, profiles 4–6). The Nb_2_O_5_-NiO/Ni-foam-C offers a single H_2_-TPR peak at 340°C with an 8°C delay compared with the NiO/Ni-foam, likely due to the weak Nb_2_O_5_-NiO interaction. The Nb_2_O_5_-NiO/Ni-foam-R delivers a main peak at 332°C and a weak shoulder at 358°C, suggesting the very limited local occurrence of moderate NiO-Nb_2_O_5_ interaction. In contrast, the Nb_2_O_5_-NiO/Ni-foam-NS provides a main peak at 371°C and a very weak one at only 297°C. It should be noted that the H_2_ consumption is attributed exclusively to the NiO reduction because Nb_2_O_5_ reduction cannot occur under such conditions (Zhang et al., 2018a, Zhang et al., 2018b). Particularly, the NiO size of the Nb_2_O_5_-NiO/Ni-foam-NS is 13.5 nm, smaller than 20 nm for the others. In general, the lattice oxygen of the smaller NiO nanocrystallites diffuses more efficiently than the larger ones (Zhu et al., 2012). So, the weak peak at 297°C is assignable to the small NiO species that interacted weakly with Nb_2_O_5_, whereas the main peak at 371°C is ascribable to the comprehensive occurrence of strong NiO-Nb_2_O_5_ interaction.

All catalysts with and without Nb_2_O_5_ modification deliver dual-peak O_2_-TPD profiles, in which the peak at 342°C is assigned to O_2_^-^ and the one at 543°C is assigned to O^−^ - (Figure 3B) (Wu et al., 2012, Iwamoto et al., 1976). The O_2_^-^ species have strong oxidizing electrophilicity and thus are considered to be non-selective oxygen species that favor the deep oxidation of product (Wu et al., 2012, Iwamoto et al., 1976). The amount and desorption behavior of O_2_^-^ and O^−^ - species are tuned markedly by Nb_2_O_5_ modification, showing clear NiO-precursor morphology dependence (Table S2 and Figure 3B). The desorbability of such two types of surface oxygen species is almost unchanged for the Nb_2_O_5_-NiO/Ni-foam-C and Nb_2_O_5_-NiO/Ni-foam-R, whereas their non-selective O_2_^-^ amounts are markedly reduced in association with a slight decline of the O^−^ amount, when compared with the Nb_2_O_5_-free samples (Table S2 and Figure 3B). For Nb_2_O_5_-NiO/Ni-foam-NS, most notably, the Nb_2_O_5_ modification makes the non-selective O_2_^-^ species almost disappear, but slightly decreases the selective O^−^ - species, whereas lowers the desorption temperature of O^−^ - species to 520°C by 23°C (Table S2 and Figure 3B). According to the Mars van Krevelen mechanism (Figure S5) (Zhu et al., 2012), the types of oxygen species determines the further reaction of ethyl radical to form ethylene (β-elimination) or CO_2_ (C-C bond cleavage). It is not surprising that Nb_2_O_5_ modification and thinning the NiO-precursor thickness are inclined to reduce the non-selective O_2_^-^ species amount and form the ethylene via β-elimination.

In nature, tuning the NiO-precursor morphology from dense Ni(OH)_2_ clump and NiC_2_O_4_ rod (450 nm) to Ni-Tp nanosheet (30 nm thickness) strengthens the NiO-Nb_2_O_5_ interaction thereby leading to almost elimination of the non-selective O_2_^-^ species and meanwhile improving the mobility of the highly selective O^−^ - species. Improved mobility of the O^−^ - species (lowered desorption temperature, Figure 3B) (Wu et al., 2012, Skoufa et al., 2014) makes it more active than the other two catalysts, which in turn compensates the activity loss caused by the reduction of non-selective O_2_^-^ and selective O^−^ - species (Zhu et al., 2016). That is the reason why the Nb_2_O_5_-NiO/Ni-foam-NS catalyst always achieves higher conversion especially above 375°C (Figure 2A).

To further gain insight into the O_2_^-^ reduction caused by Nb_2_O_5_-NiO interaction, the surfaces of the NiO/Ni-foam and Nb_2_O_5_-NiO/Ni-foam catalysts were probed by X-ray photoelectron spectroscopy (XPS). Figure 3C shows the Ni2p spectra of the catalyst samples. Three peaks are detected: main peak at binding energy (BE) of 853.8 eV for Ni^2+^ in NiO; satellite peak at 855.8 eV S(I) for Ni^3+^ in Ni_2_O_3_, Ni^2+^-OH species, and Ni^2+^ vacancies; and the other satellite peak at 861.3 S(II), involving a ligand-metal charge transfer (Salagre et al., 1996, Veenendaal and Sawatzky, 1993). The intensity ratio of S(I) to the main peak at 853.8 eV has been used to present the surface and/or structural density of defect sites (Solsona et al., 2012, Zhu et al., 2015), offering the information about the non-stoichiometric (or non-selective) property of NiO. Notably, this ratio declines from 4.0 for the NiO/Ni-foam-NS to 1.9 for the Nb_2_O_5_-NiO/Ni-foam-C, to 1.7 for the Nb_2_O_5_-NiO/Ni-foam-R, and further to 1.1 for the Nb_2_O_5_-NiO/Ni-foam-NS (Table S3). Clearly, Nb_2_O_5_ modification provides the ability to markedly reduce the non-stoichiometric Ni^3+^ (responsible for the non-selective O_2_^-^ species), whereas the nanosheet NiO-precursor morphology synergistically promoted such Nb_2_O_5_ modification effect. This observation is in good agreement with the O_2_-TPD results (Figure 3B). Figure 3D shows the XPS spectra in Nb3d region for the Nb_2_O_5_-NiO/Ni-foam catalysts. Taking the Nb^5+^ in pure Nb_2_O_5_ (207.4 eV) as reference (Liu et al., 2016), the BE of Nb^5+^ shifts to 207.2 eV for the Nb_2_O_5_-NiO/Ni-foam-C, 207.1 eV for the Nb_2_O_5_-NiO/Ni-foam-R, and then 206.9 eV for the Nb_2_O_5_-NiO/Ni-foam-NS. This trend is consistent with the increasingly stronger NiO-Nb_2_O_5_ interaction (Zhu et al., 2012).

**Figure 4:**
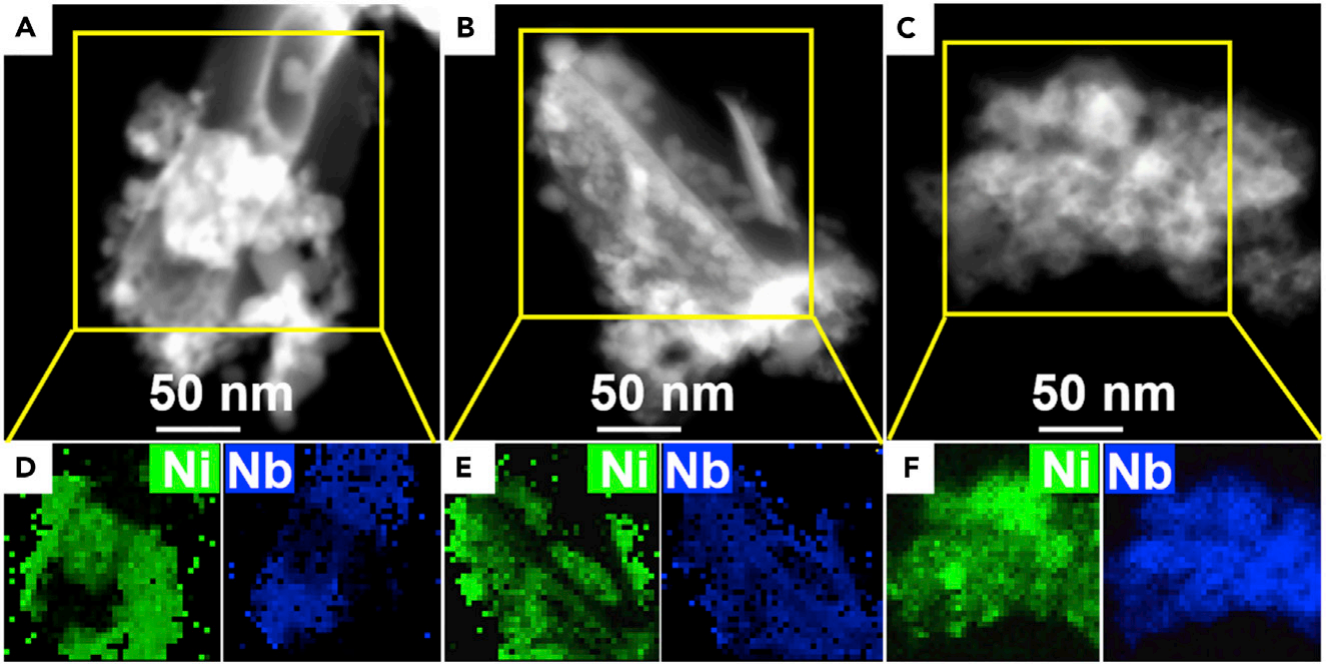
The Transmission Electron Microscopic Images of the Nb_2_O_5_-NiO/Ni-Foam Catalysts (A–F) (A–C) High-angle annular dark-field scanning transmission electron microscopic images and (D–F) elemental maps of (A and D) Nb_2_O_5_-NiO/Ni-foam-C, (B and E) Nb_2_O_5_-NiO/Ni-foam-R, and (C and F) Nb_2_O_5_-NiO/Ni-foam-NS.

As aforementioned, the nanosheet Ni-Tp precursor is much thinner than Ni(OH)_2_ clump and NiC_2_O_4_ rod and is irregularly aligned to form a porous layer (Figures 1F, 1I, and 1L). This morphology undoubtedly gives higher SSA, which is helpful for highly dispersing Nb_2_O_5_ into the NiO matrix (Figures 1S, 1T, and 1U), leading to the lower Ni/Nb ratio in catalyst surface (Table S3); furthermore, as indicated by the high-angle annular dark-field scanning transmission electron microscopy images and elemental maps in Figures 4A–4F, the Nb_2_O_5_-NiO/Ni-foam-NS achieves the contacting of NiO with Nb_2_O_5_ more sufficient than the Nb_2_O_5_-NiO/Ni-foam-C and Nb_2_O_5_-NiO/Ni-foam-R. On the other hand, the thinner nanosheet feature of Ni-Tp facilitates the incorporation of Nb ions into NiO during calcination treatment. Indeed, the lattice constant obtained by XRD (Solsona et al., 2012) reveals that the NiO lattice constant in the Nb_2_O_5_-NiO/Ni-foam-NS is 4.1724 Å, smaller than 4.1767 Å for the NiO/Ni-foam and 4.1752 Å for both the Nb_2_O_5_-NiO/Ni-foam-C and Nb_2_O_5_-NiO/Ni-foam-R (Table 1). This observation evidences that Nb ions are, at least partially, incorporated into NiO to the most extent for the Nb_2_O_5_-NiO/Ni-foam-NS (Solsona et al., 2012, Zhu et al., 2012).

### Design of the Advanced Catalyst

**Figure 5:**
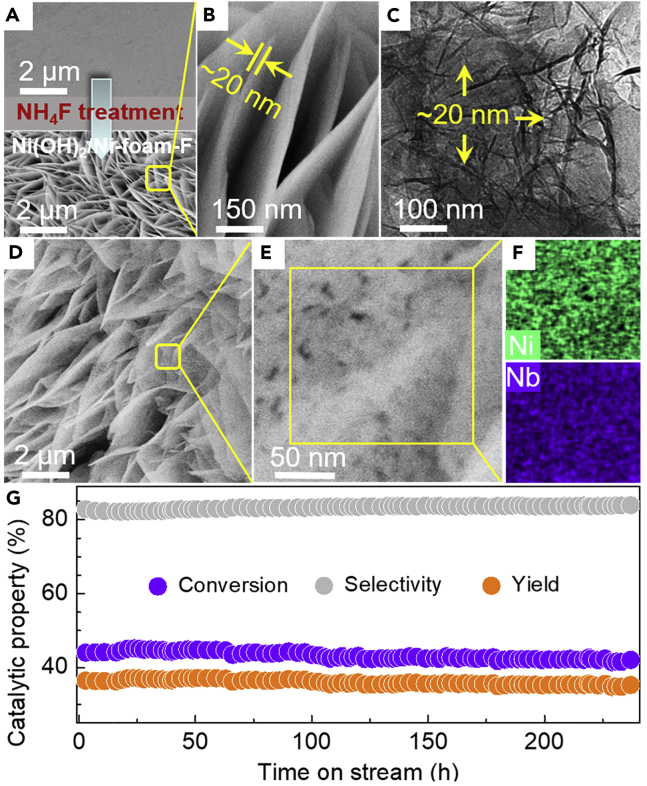
The Structure and Morphological Features of the Ni(OH)_2_/Ni-foam-F Material and the Nb_2_O_5_-NiO/Ni-foam-F Catalyst and the Stability Test of the Nb_2_O_5_-NiO/Ni-foam-F Catalyst (A–C) (A, upper) Scanning electron microscopic (SEM) image of the pristine Ni-foam. (A, lower, and B) SEM and (C) transmission electron microscopic images of Ni(OH)_2_/Ni-foam-F. (D–F) (D and E) SEM and (F) energy-dispersive X-ray images of Nb_2_O_5_-NiO/Ni-foam-F. (G) Stability testing of Nb_2_O_5_-NiO/Ni-foam-F (reaction conditions: 400°C, C_2_H_6_/O_2_/N_2_ of 1/1/8, GHSV of 9,000 cm^3^ g^−1^ h^−1^).

Last but not the least, according to the foregoing findings, we are confident that the ODE performance of Nb_2_O_5_-NiO/Ni-foam catalyst can be improved further if the NiO-precursor nanosheet is able to be thinned further. Indeed, the Ni(OH)_2_ nanosheet (∼20 nm) is successfully structured onto the Ni-foam by hydrothermal treatment in an aqueous solution of NH_4_F (denoted as Ni(OH)_2_/Ni-foam-F, Figures 5A–5C and S6), and therefore, a Nb_2_O_5_-NiO/Ni-foam-F catalyst was obtained by subsequent Nb_2_O_5_ modification. As expected, such catalyst shows much higher activity and selectivity than the Nb_2_O_5_-NiO/Ni-foam-C; when compared with the Nb_2_O_5_-NiO/Ni-foam-NS it achieves comparable activity but markedly improved selectivity (Figure S7). Notably, our Nb_2_O_5_-NiO/Ni-foam-F catalyst yields better performance (especially the selectivity, stability, and TOF) than the NiO-based catalysts (Tables 2 and S4) and powdered Nb_2_O_5_/NiO (5/21, w/w) catalyst literature (Table S5). Moreover, the ethylene yield (ethane conversion times ethylene selectivity) for such catalyst is comparable to the costly MoVTeNbO catalyst when it is tested at 2,120 cm^3^ g^−1^ h^−1^, but our catalyst runs at much higher reactor capacity (GHSV) of 9,000 cm^3^ g^−1^ h^−1^ (Table 2).

**Table 2:** Representative ODE Results for Reported NiO-Based and MoVTeNbO Catalysts

Catalyst	C_2_/O_2_/Inert Molar Ratio	Temp. (^o^C)	GHSV (cm^3^ g^−1^ h^−1^)	Conversion (%)	Selectivity (%)	TOF (h^−1^)^a^	Ref.
Nb_2_O_5_-NiO/Ni-foam-F	1/1/8	410	9,000	60	80	0.94	This work
NiNbO	1/1/8	400	6,600	65	71	0.37	Heracleous and Lemonidou (2006)
NiNbO/Al_2_O_3_	1/1/9	400	6,600	27	70	0.84	Heracleous et al., 2005
NiTaO	1/1/8	375	6,000	60	72	0.77	Zhu et al. (2015)
NiSnO	1/1/9	350	3,000	26	75	0.10	Solsona et al. (2012)
NiWO	2/2/17	400	6,000	52	60	–	Zhu et al., 2016
NiTiO	2/2/17	400	6,000	50	66	–	Zhu et al., 2016
MoVNbTeO	3/2/5	400	2,120	59	89	–	Botella et al. (2004)
	9/6/85	400	780	87	84		

a TOF is defined as the amount of ethylene formed per NiO site per hour.

In addition, it is not surprising that the Nb_2_O_5_-NiO/Ni-foam-F exhibits highly enhanced Nb_2_O_5_-NiO interaction (Figures 5D–5F) by further thinning the NiO-precursor, which results in a further reduction of the NiO lattice constant (Table 1), the NiO nanoparticle size (Table 1 and Figure S6), and especially the non-selective O_2_^-^ amount as well as the NiO reducibility (Figure S8), compared with the ones using Ni(OH)_2_/Ni-foam-C (dense clump of Ni(OH)_2_) and Ni-Tp/Ni-foam-NS (∼30 nm Ni-Tp nanosheet). This is undoubtedly responsible for the further catalytic performance improvement observed on the Nb_2_O_5_-NiO/Ni-foam-F catalyst. Most notably, this catalyst exhibits favorable stability, being stable for at least 240 h at 400°C with ∼44% ethane conversion and ∼82% ethylene selectivity (Figure 5G), which shows great superiority when compared with the previously reported Nb_2_O_5_-NiO catalysts (Table S4). This is benefited from the high Nb_2_O_5_-NiO sintering resistance (evidenced by the well-preserved SSA and particle size of NiO for the used catalyst, Table 1), as a result of the strong interaction between NiO and Nb_2_O_5_ (Solsona et al., 2011, Solsona et al., 2012) in combination with the enhanced heat transfer of the Ni-foam-structured designing that could rapidly dissipate the large quantity of reaction heat from the ODE reaction (Table S5) (Li et al., 2015, Zhao et al., 2016, Zhang et al., 2018a, Zhang et al., 2018b).

## Discussion

In summary, a low-temperature active, highly selective, and highly stable Nb_2_O_5_-NiO/Ni-foam catalyst has been developed for the ODE reaction, by carefully tuning the NiO-precursor morphology-dependent Nb_2_O_5_-NiO interaction. The Nb_2_O_5_-NiO interaction can be markedly improved by thinning the NiO-precursors endogenously grown onto the Ni-foam substrate, especially leading to significant elimination of the nonselective O_2_^-^ species and, meanwhile, remarkable improvement of the mobility of selective O^−^ species. This work provides an interesting clue to tailor high-performance ODE catalyst via morphology modulation strategy.

### Limitations of the Study

The ammonium niobium oxalate is a little bit costly.

## Methods

All methods can be found in the accompanying Transparent Methods supplemental file.

## Supplemental Information

Document S1. Transparent Methods, Figures S1–S8, and Tables S1–S5
