# Supplementary material for: Oxidative Dehydrogenation of Ethane: Superior Nb2O5-NiO/Ni-Foam Catalyst Tailored by Tuning Morphology of NiO-Precursors Grown on a Ni-Foam
Source: iScience. 2019 Sep 17;20:90–9. doi: 10.1016/j.isci.2019.09.021 (PMC6833484; doi:10.1016/j.isci.2019.09.021)
Supplement: Document S1. Transparent Methods, Figures S1–S8, and Tables S1–S5 [file mmc1.pdf]

ISCI, Volume *20*

Supplemental Information

Oxidative Dehydrogenation of Ethane: Superior Nb_2O_5-NiO/Ni-Foam Catalyst Tailored by Tuning Morphology of NiO-Precursors Grown on a Ni-Foam

Zhiqiang Zhang, Guofeng Zhao, Weidong Sun, Ye Liu, and Yong Lu

Supplemental Information

Supplemental Tables

Table S1. Number of NiO sites for the Ni-foam structured catalysts, related to Table 1.

Catalyst	Number of NiO sites ($\times 10^{19}$ g_{cat}^{-1})[a]
NiO/Ni-foam-C	7.2
NiO/Ni-foam-R	7.0
NiO/Ni-foam-NS	7.1
Nb_2O_5-NiO/Ni-foam-C	7.2
Nb_2O_5-NiO/Ni-foam-R	7.1
Nb_2O_5-NiO/Ni-foam-NS	10.1
Nb_2O_5-NiO/Ni-foam-F	10.6

[a] Calculated by using the NiO density of 6.67 g cm^{-3} and the average crystallite diameter determined by XRD.

Table S2. The quantitative analysis of O_2-TPD results, related to Figure 3.

Catalyst	O_2^- peak area	O^- peak area	O_2^-/O^- ratio	Total area
NiO/Ni-foam-C	988	931	1.06	1920
NiO/Ni-foam-R	956	903	1.06	1859
NiO/Ni-foam-NS	964	917	1.05	1881
Nb_2O_5-NiO/Ni-foam-C	505	831	0.61	1336
Nb_2O_5-NiO/Ni-foam-R	457	792	0.56	1246
Nb_2O_5-NiO/Ni-foam-NS	167	552	0.30	719

Table S3. XPS results of the as-prepared catalysts, related to Figure 3.

Catalyst	Ni/Nb ratio in surface (at./at.)	Ni $2p_{3/2}$ (eV)[a]			Intensity ratio of S(I) to Main peak
		Main peak	BE satellite		
			I	II	
NiO/Ni-foam-NS	-	853.7	855.8	861.3	4.0
Nb_2O_5-NiO/Ni-foam-C	4.3	853.8	855.7	861.2	1.9
Nb_2O_5-NiO/Ni-foam-R	4.6	853.8	855.7	861.2	1.7
Nb_2O_5-NiO/Ni-foam-NS	6.7	853.9	855.7	861.2	1.1

[a]Estimated experimental error of ±0.1 eV.

Table S4. The results of stability test reported in the literature for the ODE reaction on the Nb_2O_5-NiO catalysts, related to Figure 5.

Catalyst	C_2/O_2/inert molar ratio	Temp. (°C)	GHSV (cm^3 g^{-1} h^{-1})	Time of stability test (h)	Conv. (%)[a]	Select. (%)[a]	Conv. (%)	Select. (%)	Loss of activity (%)	Ref.
Nb_2O_5-NiO/Ni-foam-F	1/1/8	400	6000	240	44	81	42	82	4.5	This work
NiNbO	2/1/17	330	6600	70	9	84	5	88	44.4	(Zhu et al., 2012)
NiNbO	1/1/9	380	6000	200	57	63	39	76	31.6	(Zhu et al., 2015)
NiNbO	2/2/17	330	6000	50	34	79	30	80	11.8	(Savova et al., 2010)

[a]Catalytic performance after stability test of 1 h.

Table S5. Temperature-rising of the Ni-foam structured catalyst and powdered catalyst,[a] related to Figure 5.

Catalyst	Temperature (^oC)		C_2H_6 Conv. (%)	Select. (%)	
	Furnace	Bed		C_2H_4	CO_2
Nb_2O_5/NiO (100-200 μm) (5/21, wt/wt)	340	353	16.8	84.4	16.6
	350	377	30.7	80.8	19.2
	360	395	42.5	77.6	22.4
	370	410	52.1	76.1	23.9
	380	422	56.6	74.7	25.1
Nb_2O_5-NiO/Ni-foam-F	350	352	15.7	88.6	11.4
	375	379	28.0	86.1	13.9
	400	408	44.0	82.7	17.3
	410	419	52.3	81.5	18.5
	425	436	59.8	80.0	20.0

[a] Reaction conditions: $C_2H_6/O_2/N_2$ = 1/1/8, GHSV = 9000 $cm^3\ g^{-1}\ h^{-1}$.

Supplemental figures

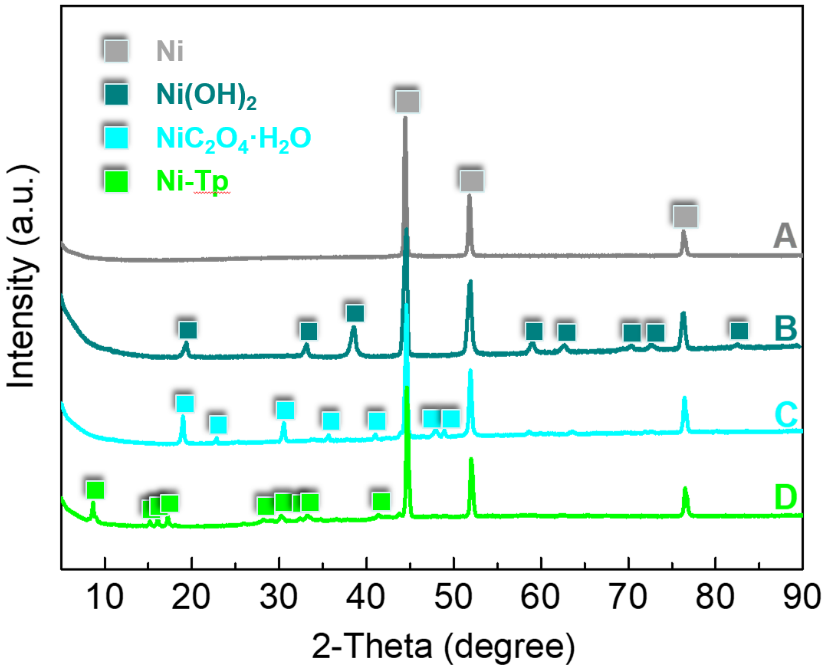

Figure S1. XRD patterns of the various NiO precursors, related to Figure 1. XRD patterns of (A) Ni-foam (showing metallic Ni diffraction peaks: 44.51°, 51.81°, 76.29°; JCPDS No. 04-0850), (B) $Ni(OH)_2$/Ni-foam (besides metallic Ni peaks, showing $Ni(OH)_2$ ones: 19.4°, 33.2°, 38.5°, 59.1°, 62.8°, 70.6°, 72.9° and 82.7°; JCPDS No. 14-0117), (C) $NiC_2O_4 \cdot 2H_2O$/Ni-foam (besides metallic Ni peaks, showing $NiC_2O_4 \cdot 2H_2O$ ones: 18.9°, 22.7°, 30.5°, 35.4°, 40.9°, 47.7° and 48.9°; JCPDS No. 01-0299), and (D) Ni-Tp/Ni-foam (besides metallic Ni peaks, showing Ni-Tp ones: 8.7°, 15.2°, 16.2°, 17.3°, 28.1°, 30.3°, 32.4°, 33.3°, 41.4° and 43.4°; JCPDS No. 35-1677).

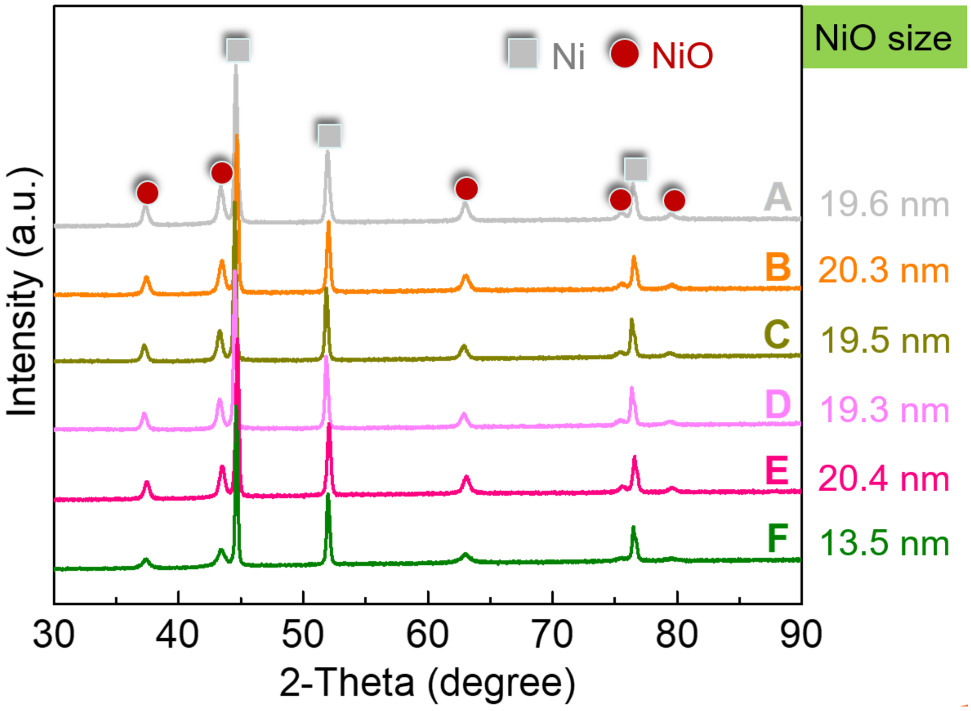

Figure S2. XRD patterns of the Ni-foam structured catalysts, related to Figure 1. XRD patterns of (A) NiO/Ni-foam-C, (B) NiO/Ni-foam-R, (C) NiO/Ni-foam-NS, (D) Nb_2O_5-NiO/Ni-foam-C, (E) Nb_2O_5-NiO/Ni-foam-R and (F) Nb_2O_5-NiO/Ni-foam-NS. *Note*: 21 wt% NiO; 5 wt% Nb_2O_5.

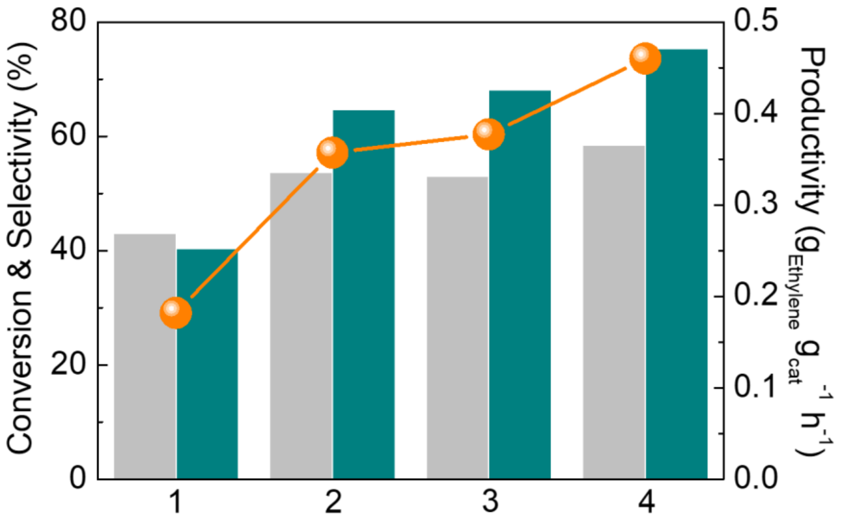

Figure S3. The ODE performance of the Ni-foam structured catalysts, **related to Figure 2.** Catalytic property of the as-prepared catalysts for ODE reaction. Note: ethane conversion (gray column), ethylene selectivity (cyan column) and productivity (orange line); 1: NiO/Ni-foam-NS; 2: Nb_2O_5-NiO/Ni-foam-C; 3: Nb_2O_5-NiO/Ni-foam-R; 4: Nb_2O_5-NiO/Ni-foam-NS. Reaction conditions: $C_2H_6/O_2/N_2$ of 1/1/8, 425 ^oC, GHSV of 9000 $cm^3\ g^{-1}\ h^{-1}$.

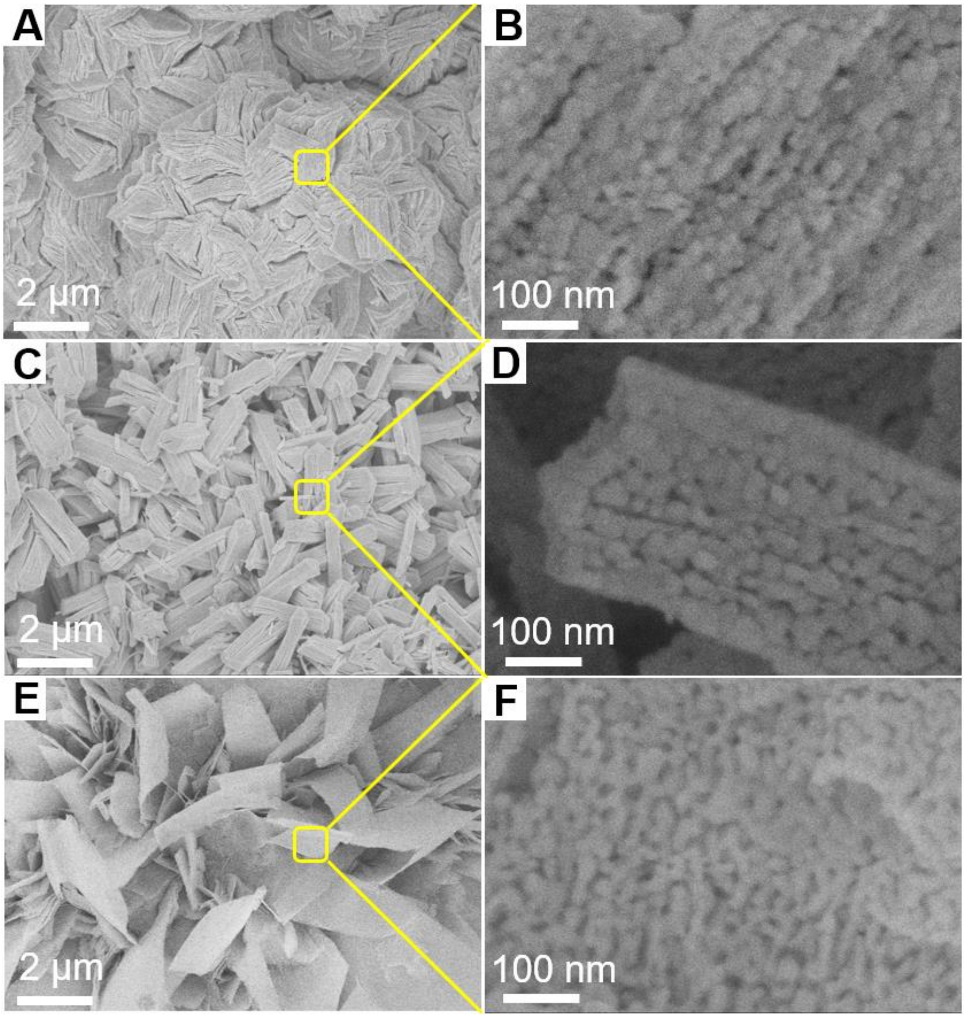

Figure S4. The structure and morphology features of the NiO/Ni-foam catalysts, related to Figure 2. SEM images of (A,B) NiO/Ni-foam-C, (C,D) NiO/Ni-foam-R and (E,F) NiO/Ni-foam-NS.

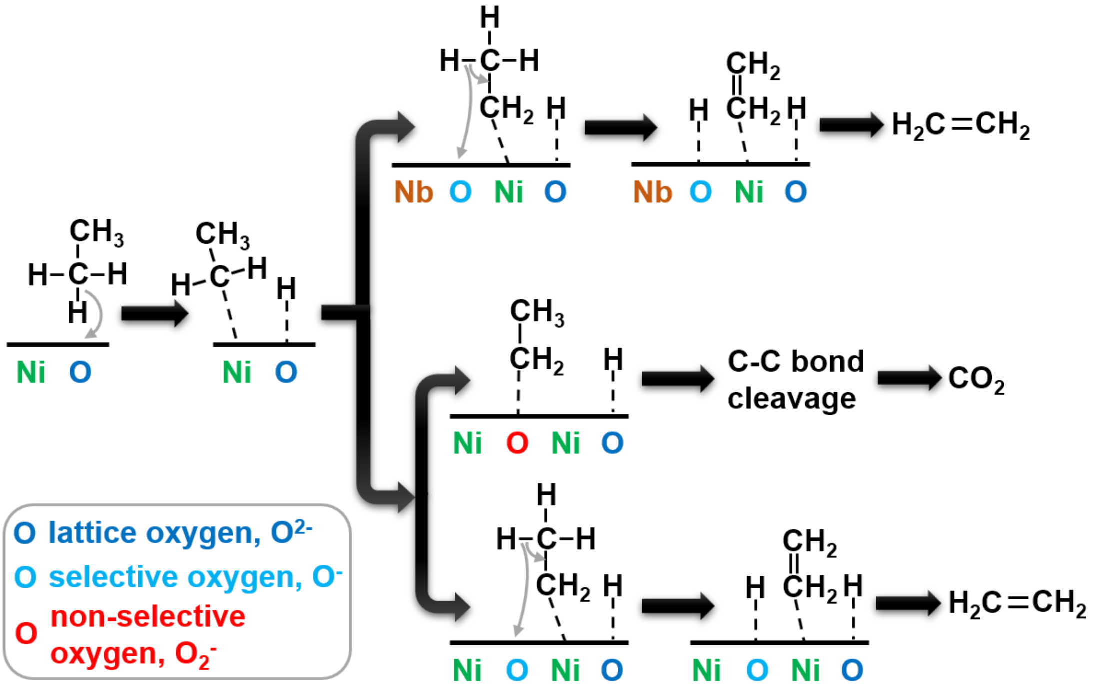

Figure S5. The schematic illustration of catalytic mechanism for ODE reaction over NiO/Ni-foam and Nb_2O_5-NiO/Ni-foam catalysts, related to Figure 2.

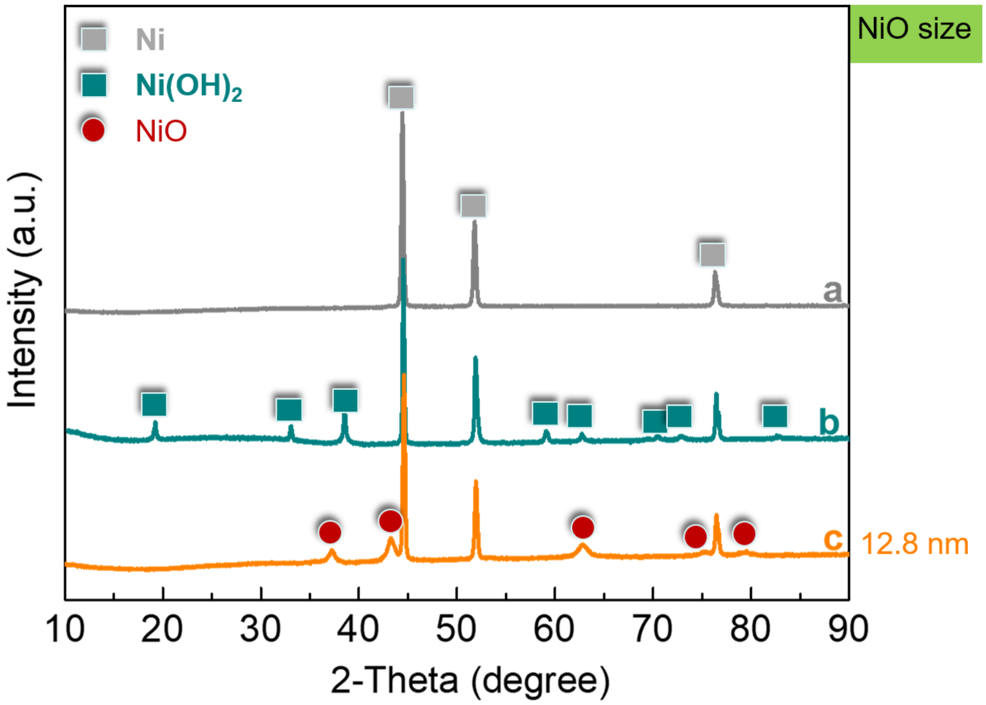

Figure S6. XRD patterns of the Ni-foam, $Ni(OH)_2$/Ni-foam-F, and Nb_2O_5-NiO/Ni-foam-F, related to Figure 5. XRD patterns of (A) Ni-foam, (B) $Ni(OH)_2$/Ni-foam-F, and (C) Nb_2O_5-NiO/Ni-foam-F.

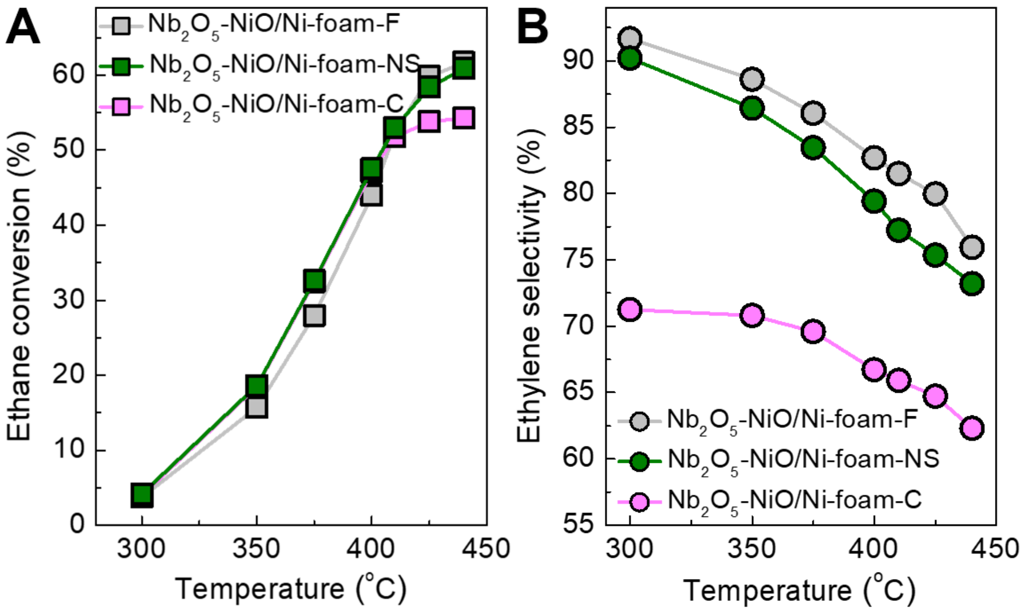

Figure S7. The ODE performance of the Ni-foam structured catalysts, related to Figure 5. Temperature-dependent (A) ethane conversion and (B) ethylene selectivity for the ODE reaction. Reaction conditions: $C_2H_6/O_2/N_2$ of 1/1/8, GHSV of 9000 cm^3 g^{-1} h^{-1}.

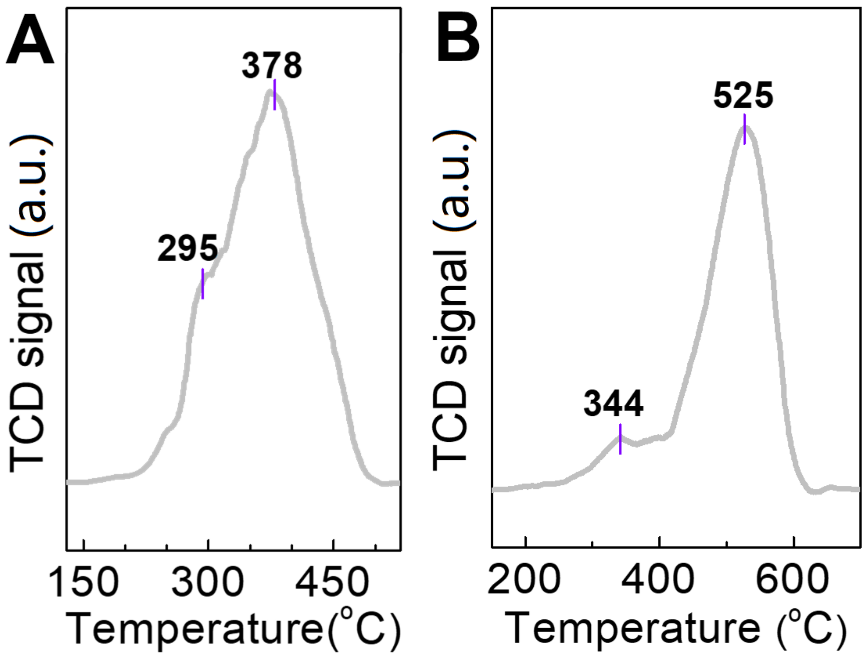

Figure S8. The characterization results of the Nb_2O_5-NiO/Ni-foam-F catalyst, related to Figure 5. (A) H_2-TPR and (B) O_2-TPD profiles for the Nb_2O_5-NiO/Ni-foam-F catalyst.

Transparent Methods

Preparation of catalysts

Pretreatment of pristine Ni-foam substrate. The pristine Ni-foam (purchased from Changsha Liyuan New Material Co. Ltd.) was carefully cleaned with 3 mol L^{-1} HCl aqueous solution in an ultrasound bath for 10 min to remove the surface NiO layer, washed with deionized water and absolute ethanol for several times, and dried at 80 ^{o}C for 12 h.

Growth of clump-like $Ni(OH)_2$ onto Ni-foam. $Ni(NO_3)_2$ $6H_2O$ of 10 mmol (2.91 g) and NH_4NO_3 of 6 mmol (0.48 g) were dissolved in deionized water of 48 mL. Then, $NH_3{\cdot}H_2O$ (28 wt%) of 6 mL was slowly added into the above solution drop by drop to obtain the nickel ammine solution. The mixed solutions were magnetically stirred for 20 min in air at room temperature. Afterwards, the as-cleaned Ni-foam (0.5 g) was immersed into the growth solution, and the in-situ growth of $Ni(OH)_2$ subsequently proceeded in water bath at 80 ^{o}C for 2 h. Finally, the resulted samples were rinsed with deionized water for several times and dried overnight at 80 ^{o}C to obtain the $Ni(OH)_2$/Ni-foam (Ye et al., 2016).

Growth of rod-like $NiC_2O_4{\cdot}2H_2O$ onto Ni-foam. Oxalic acid dihydrate of 15 mmol (1.89 g) and NH_4Cl of 1.5 mmol (0.081 g) were dissolved in deionized water of 50 mL. The solution was magnetically stirred for 20 min in air at room temperature and then transferred into a stainless steel Teflon-lined autoclave with 100 mL capacity. The as-cleaned Ni-foam (0.5 g) was immersed into the solution and then heated at 180 ^{o}C for 24 h. After the hydrothermal process, the resulted samples were rinsed with deionized water for several times and then dried overnight at 80 ^{o}C to obtain the NiC_2O_4/Ni-foam (Zhang et al., 2018).

Growth of nanosheet-like nickel terephthalate (Ni-Tp) onto Ni-foam. Terephthalic acid of 7 mmol (1.16 g) and $Ni(NO_3)_2$ $6H_2O$ of 7 mmol (2.04 g) were dissolved in 50 mL of N,N-dimethylformamide (DMF). The solution was magnetically stirred for 20 min in air at room temperature and then transferred into a stainless steel Teflon-lined autoclave with 100 mL capacity. The as-cleaned Ni-foam (0.5 g) was immersed into the solution and heated at 150 ^{o}C for 24 h. After the hydrothermal process, the resulted samples were rinsed successively with deionized water and absolute ethanol for several times and then dried overnight at 80 ^{o}C to obtain the Ni-Tp/Ni-foam (Chen et al., 2017).

Growth of nanosheet-like $Ni(OH)_2$ onto Ni-foam. $Ni(NO_3)_2$ $6H_2O$ of 4 mmol (2.33 g) and $CO(NH_2)_2$ of 20 mmol (2.40 g) were dissolved in 30 mL aqueous solution of NH_4F (containing NH_4F of 8 mmol, 0.592 g). The solution was magnetically stirred for 20 min in air at room temperature and then transferred into a stainless steel Teflon-lined autoclave with 100 mL capacity. The as-cleaned Ni-foam (0.5 g) was immersed into the solution and heated at 100 ^{o}C for 12 h. After the hydrothermal process, the resulted samples were rinsed successively with deionized water several times and then dried overnight at 80 ^{o}C to obtain the $Ni(OH)_2$/Ni-foam-F (Huang et al., 2014).

Preparation of NiO/Ni-foam catalysts. The as-obtained $Ni(OH)_2$/Ni-foam, NiC_2O_4/Ni-foam, Ni-Tp/Ni-foam, and $Ni(OH)_2$/Ni-foam-F samples were calcined in air at 450 ^{o}C for 2 h to form the NiO/Ni-foam-C, NiO/Ni-foam-R, NiO/Ni-foam-NS, and NiO/Ni-foam-F catalysts, respectively.

Preparation of Nb_2O_5-NiO/Ni-foam catalysts. The as-obtained $Ni(OH)_2$/Ni-foam, NiC_2O_4/Ni-foam, Ni-Tp/Ni-foam, and $Ni(OH)_2$/Ni-foam-F samples were directly impregnated

with an aqueous solution of ammonium niobium oxalate of specific concentration in the incipient wetness manner. After drying at 100 ^{o}C overnight and calcining in air at 450 ^{o}C for 2 h, the Nb_2O_5-NiO/Ni-foam-C, Nb_2O_5-NiO/Ni-foam-R, Nb_2O_5-NiO/Ni-foam-NS, and Nb_2O_5-NiO/Ni-foam-F catalysts were obtained, respectively. Note that the loadings of NiO and Nb_2O_5 could be tuned via controlling the preparation conditions of different NiO precursors and added amount of ammonium niobium oxalate, respectively.

Preparation of the powdered Nb_2O_5-NiO catalyst. The nanosheet-like $Ni(OH)_2$ powders were obtained by solvothermal method using the solution of $Ni(NO_3)_2$, $CO(NH_2)_2$ and NH_4F in 100 ^{o}C for 12 h (Huang et al., 2014). The powdered catalyst Nb_2O_5/NiO (5/21, wt/wt; 100-200 μm) was prepared by impregnation of nanosheet-like $Ni(OH)_2$ powders with niobium ammonium oxalate solution followed by calcination in air at 450 ^{o}C.

Characterization of catalysts

Crystalline phases of all catalysts were probed by X-ray diffraction (XRD, Rigaku Ultima IV) in the 2θ scanning range of 20-90° at a scanning rate of 10°/min, using the Cu Kα radiation source generated at 30 kV and 25 mA. Particle size of NiO was calculated by the Scherrer equation at 2θ of 43.3^{o}. N_2 adsorption/desorption isotherms were measured at 77 K on a BEL-MAX gas adsorption analyzer. Prior to the measurement, the samples were degassed for 6 h at 100 ^{o}C before nitrogen adsorption. Specific surface area (SSA) was calculated from the adsorption branch in the range of relative pressure from 0.05 to 0.25 by Brunauer-Emmett-Teller (BET) method. Catalyst geometry and morphology were characterized by scanning electron microscopy (SEM, Hitachi S-4800, Japan) with an energy dispersive X-ray spectrometry (EDX) and transmission electron microscopy (TEM, FEI-Tecnai G2F30). Reducibility of NiO species for the catalysts was analyzed by H_2-temperature programmed reduction (H_2-TPR) while the NiO content was calculated on the basis of H_2 consumption data deduced from H_2-TPR experiments (Li et al., 2015). Distribution and amount of surface oxygen species for the catalysts was analyzed by O_2-temperature programmed desorption (O_2-TPD). H_2-TPR and O_2-TPD experiments were all performed on a TP 5080 multi-functional automatic adsorption instrument (Xianquan Industrial and Trading Co., Ltd) with a thermal conductivity detector (TCD). For each H_2-TPR trial, sample (100 mg) purged by He at 450 ^{o}C for 30 min in advance was heated from 25 to 600 ^{o}C in a gas mixture of 10% H_2 in N_2 (30 mL min^{-1}) at a heating ramp of 10 ^{o}C min^{-1}. For each O_2-TPD trial, sample (200 mg) was treated at 450 ^{o}C for 30 min and cooled down to room temperature (r.t.) in a 3 vol % O_2/He flow (30 mL min^{-1}). After that, the sample flushed in He carrier flow (30 mL min^{-1}) until a horizontal TCD baseline appeared was heated from r.t. to 850 ^{o}C at a heating rate of 15 ^{o}C min^{-1}. The non-stoichiometric property of the catalysts was analyzed by X-ray photoelectron spectroscopy (XPS), which was recorded on an Escalab 250xi spectrometer, using a standard Al Kα X-ray source (300 W) and an analyzer pass energy of 20 eV. All binding energies were referenced to the adventitious C1s line at 284.6 eV.

ODE Reaction Test

The ODE reaction with molecular oxygen was performed in a fixed-bed quartz tube reactor

(i.d., 8 mm; reactor length of 700 mm) under atmospheric pressure using a gas hourly space velocity (GHSV) of 9,000 cm^3 g^{-1} h^{-1}. Circular chips (8.1 mm diameter) of the catalysts were packed layer-by-layer into the tube reactor with and the catalyst dosage was 0.2 g. Note that the diameter of 0.1 mm larger than the i.d. of the tubular reactor was retained deliberately to avoid the appearance of gap between the reactor wall and the edges of catalyst chips thereby preventing the gas bypassing. In addition, the powdered Nb_2O_5/NiO catalyst of 52 mg (i.e., equivalent amount of Nb_2O_5 plus NiO to that for the Nb_2O_5-NiO/Ni-foam-F catalyst) was dosed into reactor, and was diluted using quartz sand to obtain the equivalent bed volume to that for the Nb_2O_5-NiO/Ni-foam-F catalyst. The catalyst bed was then heated from r.t. to the reaction temperature ranged from 300 to 450 ^{o}C in a gaseous $C_2H_6/O_2/N_2$ mixture (molar ratio of 1/1/8). Three calibrated mass flow controllers were used to control the oxygen, nitrogen and ethane gas, of which the purity is > 99.99%. Effluent gas was analyzed by an online HP 6850 gas chromatograph equipped with a thermal conductivity detector (TCD) connected to Plot U and MS 5A parallel capillary columns (DIKMA). A Plot U column was used to separate CO_2, C_2H_4, and C_2H_6 and a 5A molecular sieve column was used to separate O_2, N_2, CH_4, and CO. Notably, CO_2 was the only byproduct detected and no CO was observed owing to the CO oxidation to CO_2 (Han et al., 2014) under our conditions. No coke was found on the catalyst during the ODE reaction test. Moreover, the mass balance calculated on the basis of carbon lies in 98-100%.

TOF Calculation

In order to further assess the intrinsic catalytic activity, so called turnover frequency (TOF) which is defined as the amount of ethylene formed per NiO site per hour (Solsona et al., 2012) are calculated and C_2H_6 conversion was controlled to be <5 % at 300 ^{o}C. The theoretical specific surface area (SSA) of catalyst is expressed as S, the area of one NiO-unity is expressed as S(NiO) and the theoretical total surface area of catalyst is expressed as S_{Total}. The a is the NiO loading and the NiO density (ρ(NiO)) is 6.67 g cm^{-3} (Zhu et al., 2015). In addition, b is the half of NiO lattice constant and $F(C_2H_6)$ is the flow of C_2H_6 (L/h). Accordingly, the theoretical number of NiO sites (N(NiO)) and TOF can be expressed as follows, assuming that the NiO hemispheres are exposed on catalyst surface.

$$S = \frac{S_{Total}}{m(Cat)} = \frac{\dfrac{\dfrac{a * m(Cat)}{\rho(NiO)}}{\dfrac{1}{2} * \dfrac{4}{3} * \pi * r(NiO)^3} * \dfrac{1}{2} * 4 * \pi * r(NiO)^2}{m(Cat)} = \frac{3 * a}{r(NiO) * \rho(NiO)}$$

$$N(NiO) = \frac{S}{S(NiO)} = \frac{\dfrac{3 * a}{r(NiO) * \rho(NiO)}}{\pi * b^2} = \frac{3 * a}{\pi * b^2 * r(NiO) * \rho(NiO)}$$

$$TOF = \frac{Yield(C_2H_4) * N(C_2H_6)}{N(NiO)} = \frac{Conv(C_2H_6) * Sel(C_2H_4) * n(C_2H_6) * N_A}{\dfrac{3 * a}{\pi * b^2 * r(NiO) * \rho(NiO)}}$$

$$= \frac{Conv(C_2H_6) * Sel(C_2H_4) * P * F(C_2H_6) * N_A * \pi * b^2 * r(NiO) * \rho(NiO)}{3 * a * R * T}$$

Supplementary References

Chen, Q., Lei, S., Deng, P., Ou, X., Chen, L., Wang, W., Xiao, Y., and Cheng, B. (2017). Direct growth of nickel terephthalate on Ni foam with large mass-loading for high-performance supercapacitors. J. Mater. Chem. A *5*, 19323-19332.

Chu, B., An, H., Nijhuis, T.A., Schouten C., and Cheng, Y. (2015). A self-redox pure-phase M1 $MoVNbTeO_x/CeO_2$ nanocomposite as a highly active catalyst for oxidative dehydrogenation of ethane. J. Catal. *329*, 471-478.

Han, S.W., Kim, D.H., Jeong, M.G., Park, K.J., and Kim, Y.D. (2016). CO oxidation catalyzed by NiO supported on mesoporous Al_2O_3 at room temperature. Chem. Eng. J. *283*, 992-998.

Heracleous, E., and Lemonidou, A.A. (2006). Ni-Nb-O mixed oxides as highly active and selective catalysts for ethene production via ethane oxidative dehydrogenation. Part I: Characterization and catalytic performance. J. Catal. *237*, 162-174.

Heracleous, E., Lee, A.F., Wilson, K., and Lemonidou, A.A. (2005). Investigation of Ni-based alumina-supported catalysts for the oxidative dehydrogenation of ethane to ethylene: structural characterization and reactivity studies. J. Catal. *231*, 159-171.

Huang, M., Li, F., Ji, J., Zhang, Y., Zhao, X., and Gao, X. (2014). Facile synthesis of single-crystalline NiO nanosheet arrays on Ni foam for high-performance supercapacitors. CrystEngComm *16*, 2878-2884.

Li, Y.K., Zhang, Q.F., Chai, R.J., Zhao, G.F., Liu, Y., and Lu, Y. (2015). Structured Ni-CeO_2-Al_2O_3/Ni-foam catalyst with enhanced heat transfer for substitute natural gas production by syngas methanation. ChemCatChem *7*, 1427-1431.

Savova, B., Loridant, S., Filkova, D., and Millet, J.M. (2010). Ni-Nb-O catalysts for ethane oxidative dehydrogenation. Appl. Catal. A *390*, 148-157.

Solsona, B., Concepción, P., Demicol, B., Hernández, S., Delgado, J.J., Calvino, J.J., and López Nieto, J.M. (2012). Selective oxidative dehydrogenation of ethane over SnO_2-promoted NiO catalysts. J. Catal. 295, 104-114.

Ye, K., Zhang, H., Zhao, L., Huang, X., Cheng, K., Wang, G., and Cao, D. (2016). Facile preparation of three-dimensional $Ni(OH)_2$/Ni foam anode with low cost and its application in a direct urea fuel cell. New J. Chem. *40*, 8673-8680.

Zhang, Z., Zhao, G., Chai, R., Zhu, J., Liu, Y., and Lu, Y. (2018). Low-temperature, highly selective, highly stable Nb_2O_5-NiO/Ni-foam catalyst for the oxidative dehydrogenation of ethane. Catal. Sci. Technol. *8*, 4383-4389.

Zhu, H., Dong, H., Laveille, P., Saih, Y., Caps, V., and Basset, J. (2014). Metal oxides modified NiO catalysts for oxidative dehydrogenation of ethane to ethylene. Catal. Today *228*, 58-64.

Zhu, H., Rosenfeld, D., Anjum, D., Sangaru, S., Saih, Y., Ould-Chikh, S., and Basset, J. (2015). Ni-Ta-O mixed oxide catalysts for the low temperature oxidative dehydrogenation of ethane to ethylene. J. Catal. *329*, 291-306.

Zhu, H.B., Ould-Chikh, S., Anjum, D.H., Sun, M., Biausque, G., Basset, J.M., and Caps, V. (2012). Nb effect in the nickel oxide-catalyzed low-temperature oxidative dehydrogenation of ethane. J. Catal. *285*, 292-303.

Zhu, H.B., Rosenfeld, D.C., Anjum, D.H., Caps, V., and Basset, J.M. (2015). Green synthesis of Ni-Nb oxide catalysts for low-temperature oxidative dehydrogenation of ethane. ChemSusChem *8*, 1254-1263.
